# Self-assembly of C_60_ on a ZnTPP/Fe(001)–*p*(1 × 1)O substrate: observation of a quasi-freestanding C_60_ monolayer

**DOI:** 10.3762/bjnano.13.76

**Published:** 2022-08-30

**Authors:** Guglielmo Albani, Michele Capra, Alessandro Lodesani, Alberto Calloni, Gianlorenzo Bussetti, Marco Finazzi, Franco Ciccacci, Alberto Brambilla, Lamberto Duò, Andrea Picone

**Affiliations:** 1 Dipartimento di Fisica, Politecnico di Milano, Piazza Leonardo da Vinci 32, 20133 Milano, Italy

**Keywords:** fullerene, scanning tunneling microscopy, ultraviolet photoemission spectroscopy, ZnTPP

## Abstract

Fullerene (C_60_) has been deposited in ultrahigh vacuum on top of a zinc tetraphenylporphyrin (ZnTPP) monolayer self-assembled on a Fe(001)–*p*(1 × 1)O substrate. The nanoscale morphology and the electronic properties of the C_60_/ZnTPP/Fe(001)–*p*(1 × 1)O heterostructure have been investigated by scanning tunneling microscopy/spectroscopy and ultraviolet photoemission spectroscopy. C_60_ nucleates compact and well-ordered hexagonal domains on top of the ZnTPP buffer layer, suggesting a high surface diffusivity of C_60_ and a weak coupling between the overlayer and the substrate. Accordingly, work function measurements reveal a negligible charge transfer at the C_60_/ZnTPP interface. Finally, the difference between the energy of the lowest unoccupied molecular orbital (LUMO) and that of the highest occupied molecular orbital (HOMO) measured on C_60_ is about 3.75 eV, a value remarkably higher than those found in fullerene films stabilized directly on metal surfaces. Our results unveil a model system that could be useful in applications in which a quasi-freestanding monolayer of C_60_ interfaced with a metallic electrode is required.

## Introduction

Vertical heterostructures composed by organic molecules interfaced with metallic substrates have been the subject of intense experimental and theoretical investigation during the last two decades [1–3]. The interest in these hybrid systems has been boosted by their applications in new emerging fields, such as nanoscale catalysis [4–5], organic electronics [6–7], and spintronics [8–9], to name just a few. From a fundamental point of view, well-defined organic/inorganic heterostructures represent an interesting benchmark for the investigation of the boundary between materials possessing antithetic electronic and structural properties. In this frame, the molecule–metal interaction arising at the interface plays a crucial role in determining the morphology and the electronic properties of the hybrid organic/inorganic system. With regard to the structural aspects, a compact molecular film is crucial to obtain high-performance devices, since an efficient charge carrier transport is hindered by morphological defects, such as grain boundaries or pinholes [10–11]. Moreover, crystalline and well-ordered layers are particularly suitable for spatially averaging measurements and for modeling by ab initio calculations. Periodic and compact films are generally obtained when the molecules possess enough surface mobility, that is, when the diffusion energy (*E*_d_) is low compared to the thermal energy *k*_B_*T*, where *T* is the substrate temperature and *k*_B_ is the Boltzmann constant [12]. Annealing the substrate during the film deposition could promote the growth of ordered layers even for high *E*_d_ values (*E*_d_ > *k*_B_*T*, with *T* = 300 K), but often the high annealing temperature required promotes the modification of the molecules or even their decomposition [13–14].

Another important aspect is the electronic coupling between the molecules and the metallic substrate. In this case, the key parameter is the adsorption energy (*E*_a_), which is defined as the energy required to desorb a molecule from the surface. A high *E*_a_ is characteristic of molecules chemisorbed on the substrate, where a relevant charge transfer between the overlayer and the substrate occurs. In contrast, a low *E*_a_ is characteristic of physisorbed molecules, for which the adsorption is mediated by the weak van der Waals interaction with the substrate.

Chemisorption is the typical scenario for molecules stabilized on metallic substrates. Here, the hybridization between the molecular orbitals and the electronic states of the substrate generally modifies the intrinsic properties of the molecules, inducing the broadening of the molecular resonances, the narrowing of the band gap, and the development of interface states [15–16]. The ability to tailor the degree of electronic coupling between the molecules and the substrate is of utmost importance when it comes to embedding the interface in a specific application. For instance, if the molecules are interfaced with ferromagnetic electrodes in spin-valve architectures, the hybridization between the electronic states of the metallic substrate and the molecular orbitals is crucial to induce spin-polarized molecular states at the organic/inorganic interface [17–19]. Conversely, if either isolated molecules or self-assembled monolayers are adsorbed on solid surfaces for the investigation of their intrinsic properties, the minimization of the molecule–substrate interaction is desirable [20]. Furthermore, a weak molecule/metal electronic coupling is required in organic solar cells, because metallic states promote the relaxation of photo-excitations, lowering the cell efficiency [21].

It has been shown that a buffer layer interposed between the substrate and the molecular film can improve the crystallinity of the latter and reduce the electronic coupling with the support [22]. The buffer layer can either be a thin oxide film [23–26] or a single layer of 2D material, such as graphene [27–28], hexagonal boron nitride [29–31] and MoS_2_ [32–33]. Moreover, an organic layer inserted between the substrate and the overlayer has been shown to be effective in improving the order of the molecular film [34–35] or restoring its original electronic structure [36–38]. In this paper, we investigate the effects induced by a ZnTPP buffer layer covering the Fe(001)–*p*(1 × 1)O surface on the electronic and structural properties of a C_60_ ultrathin film. The Fe(001)–*p*(1 × 1)O surface is characterized by a single layer of oxygen atoms, adsorbed in the hollow sites of the Fe(001) surface [39–41]. The deposition of a single layer of ZnTPP on Fe(001)–*p*(1 × 1)O leads to the stabilization of a well-ordered organic film, forming a (5 × 5) superstructure with respect to the substrate [42–46]. It is important to notice that the deposition of ZnTPP directly on the bare Fe(001) surface results in a completely disordered film [47], therefore the passivation of Fe(001) with oxygen is a crucial step to obtain a suitable molecular buffer layer. Since porphyrins molecules lie flat on the Fe(001)–*p*(1 × 1)O surface, the ZnTPP wetting layer provides an ideal buffer layer for the growth of C_60_, which forms a compact film weakly coupled with the metallic substrate.

## Materials and Methods

The experiments were performed in two ultrahigh vacuum (UHV) systems. Clean Fe(001) is obtained by deposition of a thick Fe film (500 nm) by molecular beam epitaxy in UHV on a MgO(001) single crystal [48]. The Fe(001)–*p*(1 × 1)O surface was prepared by using the following procedure: the clean Fe substrate was exposed to 30 Langmuir of molecular oxygen at a pressure of 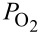 = 2 × 10^−7^ mbar and subsequently annealed at about 700 °C for 5 min. Porphyrins were sublimated by Knudsen effusion cells. The deposition flux was 0.5 ML/min, with 1 ML = 3.06 Å, as monitored by a quartz microbalance. C_60_ was evaporated on top of 1 ML ZnTPP/Fe(001)–*p*(1 × 1)O. STM images have been acquired at room temperature in constant-current mode with custom-made electrochemically etched W tips.

Scanning tunneling spectroscopy (STS) data, that is, d*I*/d*V* curves for the investigation of the sample density of states (DOS), have been collected at room temperature, using a lock-in amplifier with a modulation amplitude of 60 mV.

All STM and STS measurements have been carried out while keeping the sample grounded and applying a continuous or sinusoidal bias voltage to the tip. We followed the convention to indicate as positive the bias for which electrons tunnel from filled states of the tip to empty states of the sample.

The ultraviolet photoelectron spectroscopy (UPS) data have been acquired at normal emission with a 150 mm hemispherical electron analyzer from SPECS GmbH. The probing depth of UPS is a few angstroms [49]. A He lamp has been employed as a source of non-monochromatized unpolarized UV photons. The He-I line, with a photon energy of 21.2 eV, has been used to excite the sample. The full width at half maximum (FWHM) energy resolution of the UPS experiment is 0.05 eV.

## Results and Discussion

**Figure 1:**
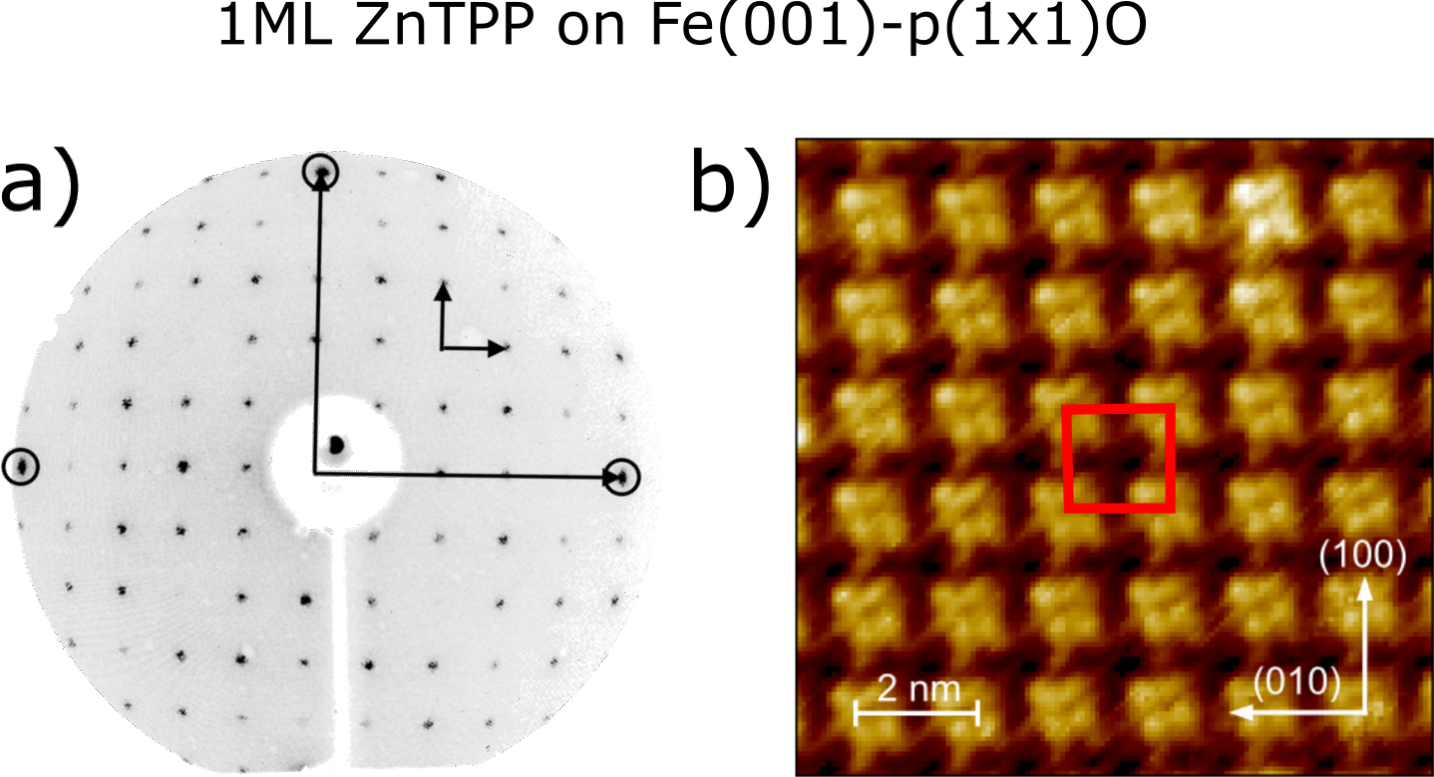
(a) LEED pattern of the system 1 ML ZnTPP /Fe(001)–*p*(1 × 1)O acquired with a beam energy equal to 55 eV. In the circles, the spots from the bare Fe(001)–*p*(1 × 1)O surface are highlighted. The arrows indicate the unit vectors in the reciprocal space of the substrate (long arrows) and after the deposition of the organic film (short arrows). (b) STM image of the ZnTPP overlayer. Tunneling parameters *V* = 1.5 V, *I* = 500 pA, image size 11 × 11 nm^2^. The red square indicates the (5 × 5) unit cell. In the lower right corner, the crystallographic directions are indicated.

Figure 1a and Figure 1b report the structural characterization of the ZnTPP/Fe(001)–*p*(1 × 1)O sample in the reciprocal and in direct space, respectively. The low-energy electron diffraction (LEED) pattern acquired on the ZnTPP/Fe(001)–*p*(1 × 1)O sample is characterized by a well-defined square lattice, where several diffraction orders are visible. Intense spots corresponding to the square lattice of the Fe(001)–*p*(1 ×1)O surface are marked with circles on the periphery of the screen. The coexisting LEED patterns of the Fe(001)–*p*(1 × 1)O surface and of the ZnTPP film allow for the quantitative evaluation of the overlayer lattice constant, which indicates that the molecules arrange themselves in a (5 × 5) commensurate array with respect to the Fe(001)–*p*(1 ×1)O surface, in agreement with previous results [46]. This order extends over large domains (hundreds of square nanometers wide) and tends to disappear as soon as additional molecules are deposited on top of the wetting layer. The formation of a well-ordered ZnTPP film with (5 × 5) periodicity is confirmed by the STM image displayed in Figure 1b, where individual ZnTPP molecules are resolved.

**Figure 2:**
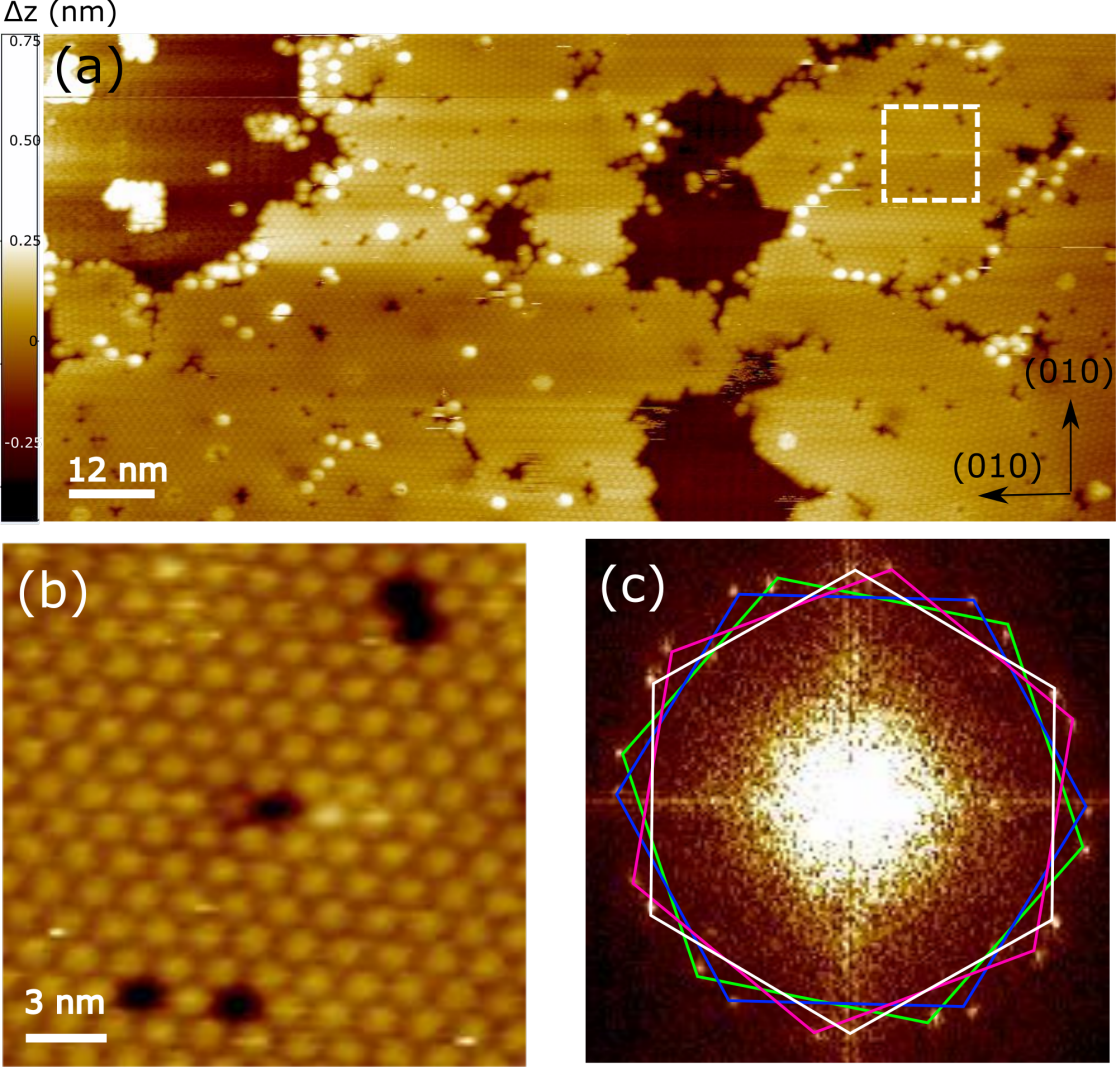
(a) Large-scale STM image of a C_60_ wetting layer deposited on C_60_/Zn-TPP/Fe(001)-*p*(1 × 1)O. In the left top corner of the image, the ZnTPP layer is visible. (b) Zoomed image of the region marked by a dashed square in panel (a). (c) FFT of the image in panel (a), where four differently oriented hexagonal domains are marked. The rotation angles between the white and the pink, blue, and green hexagonal domains are 10°, 33°, and 44°, respectively. STM images have been acquired at *V* = 1.5 V and *I* = 500 pA.

Figure 2 focuses on the surface morphology for a sub-monolayer coverage of C_60_ on the ZnTPP/Fe(001)–*p*(1 × 1)O substrate. C_60_ forms a compact film, composed of hexagonal domains extending for hundreds of nanometers. By considering that the deposition has been performed with the substrate kept at room temperature, we can estimate that *E*_d_ for C_60_ diffusing on ZnTPP is significantly lower than 25 meV. It is worth to notice that the ZnTPP buffer layer remarkably decreases *E*_d_ with respect to the case of C_60_ deposited at room temperature directly on either the Fe(001) or Fe(001)–*p*(1 × 1)O surfaces. In the former case, the diffusion of C_60_ is completely hindered and fullerene forms a disordered film, while in the latter case a peculiar mode of growth, intermediate between diffusion-mediated and ballistic growth, is observed [23,50]. Figure 2b shows a blowup of one fullerene domain, where individual C_60_ molecules are visible inside a hexagonal lattice with a lattice parameter of about 1 nm, a value very similar to that measured in C_60_ films stabilized on either metallic [51] or oxide [25] substrates. Figure 2c shows the fast Fourier transform (FFT) calculated from the image reported in Figure 2a. Four hexagonal domains can be identified, differing by their angular orientation with respect to the substrate. Interestingly, the domains do not possess a well-defined epitaxial relation with respect to the (5 × 5) lattice of ZnTPP, indicating a weak interaction between the C_60_ film and the ZnTPP substrate, as confirmed by the spectroscopic measurements presented in the following.

**Figure 3:**
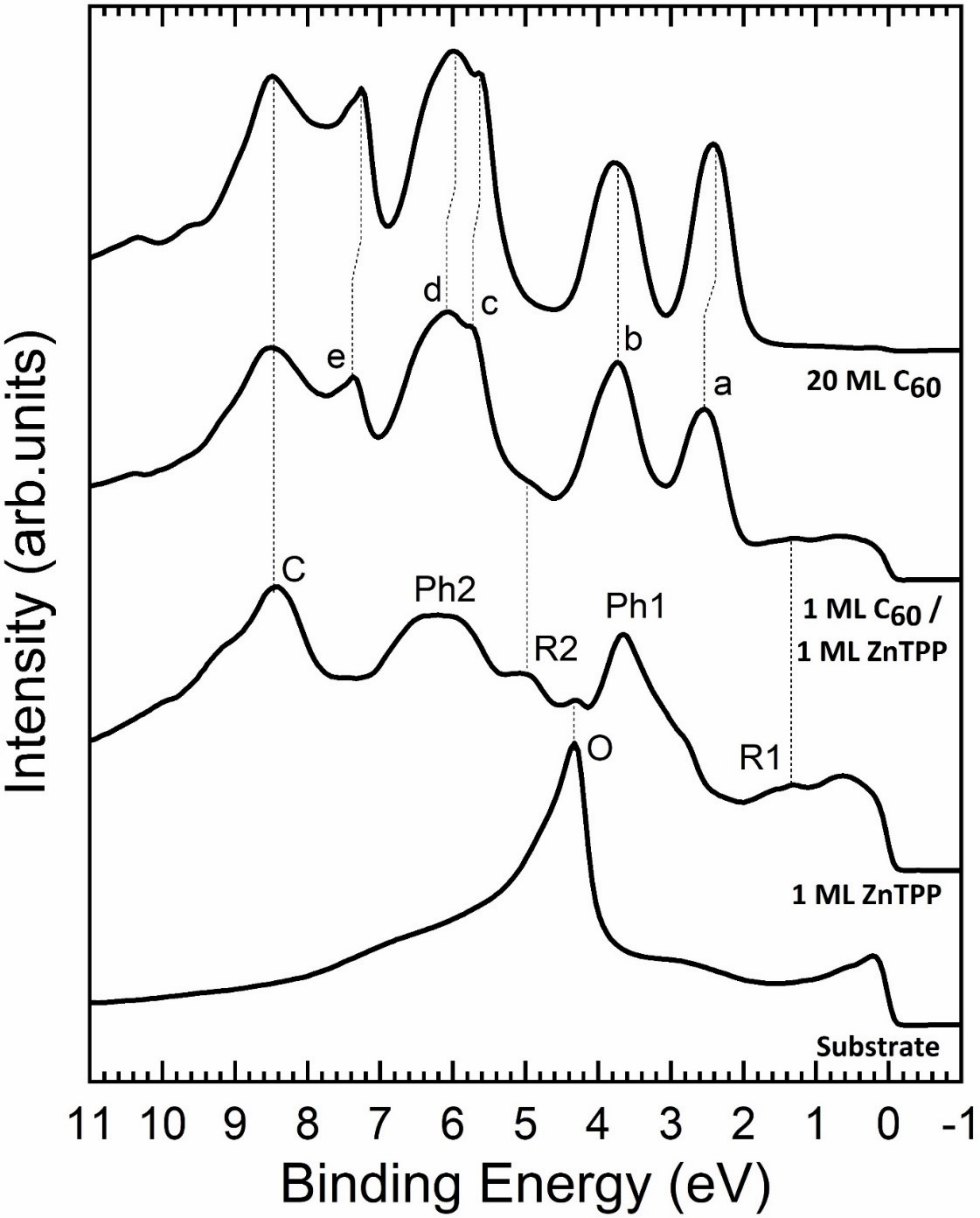
UPS spectra of the system Fe(001)–*p*(1 × 1)O at different coverages of ZnTPP and C_60_. The lowest spectrum is the one from the bare Fe(001)–*p*(1 × 1)O. The main features from Fe(001)–*p*(1 × 1)O (the peak due to oxygen, “O”), ZnTPP (both from the pyrrolic macroring, “R1” and “R2”, and from the phenyl subunits, “Ph1” and “Ph2”) and from C_60_ (“a”–“e”) are labeled and their evolution is indicated with dotted lines.

The UPS spectra acquired on Fe(001)–*p*(1 × 1)O, ZnTPP Fe(001)–*p*(1 × 1)O, 1 ML C_60_/ZnTPP/Fe(001)–*p*(1 × 1)O, and 20 ML C_60_/Fe(001)–*p*(1 × 1)O samples are shown in Figure 3. The spectrum of Fe(001)–*p*(1 × 1)O is dominated by a large peak located at about 4.2 eV, which is attributed to O 2p states. This feature almost completely vanishes as soon as 1 ML of ZnTPP is deposited, indicating that oxygen remains buried at the ZnTPP/Fe(001)–*p*(1 × 1)O interface. In the 1 ML ZnTPP spectrum in Figure 3, the UPS peaks related to the main molecule ring and to the phenyl groups are labeled “R” and “Ph” [52–53], respectively, according to theoretical simulations performed on metal tetraphenyl porphyrins and metal porphyrins [54]. When an additional single layer of C_60_ is added to this system, new features appear. The photoemission signal from the underlying ZnTPP layer, albeit affected by the screening action of C_60_ (implying a rather large surface sensitivity of the technique, as also shown in [55] on a similar system), is still detected in those spectral regions not superimposed to the new C_60_ features. In particular, peaks “a” and “b” can be readily assigned to HOMO and HOMO−1 features and their energetic positions match with their equivalents when a very thick layer of C_60_ is grown directly on Fe(001)–*p*(1 × 1)O (top spectrum). The feature labeled “c” in Figure 3 is due to C 2p electrons [56]. Therefore, it is present with only slight modifications both in ZnTPP/Fe(001)–*p*(1 × 1)O and C_60_/ZnTPP/Fe(001)–*p*(1 × 1)O samples.

**Figure 4:**
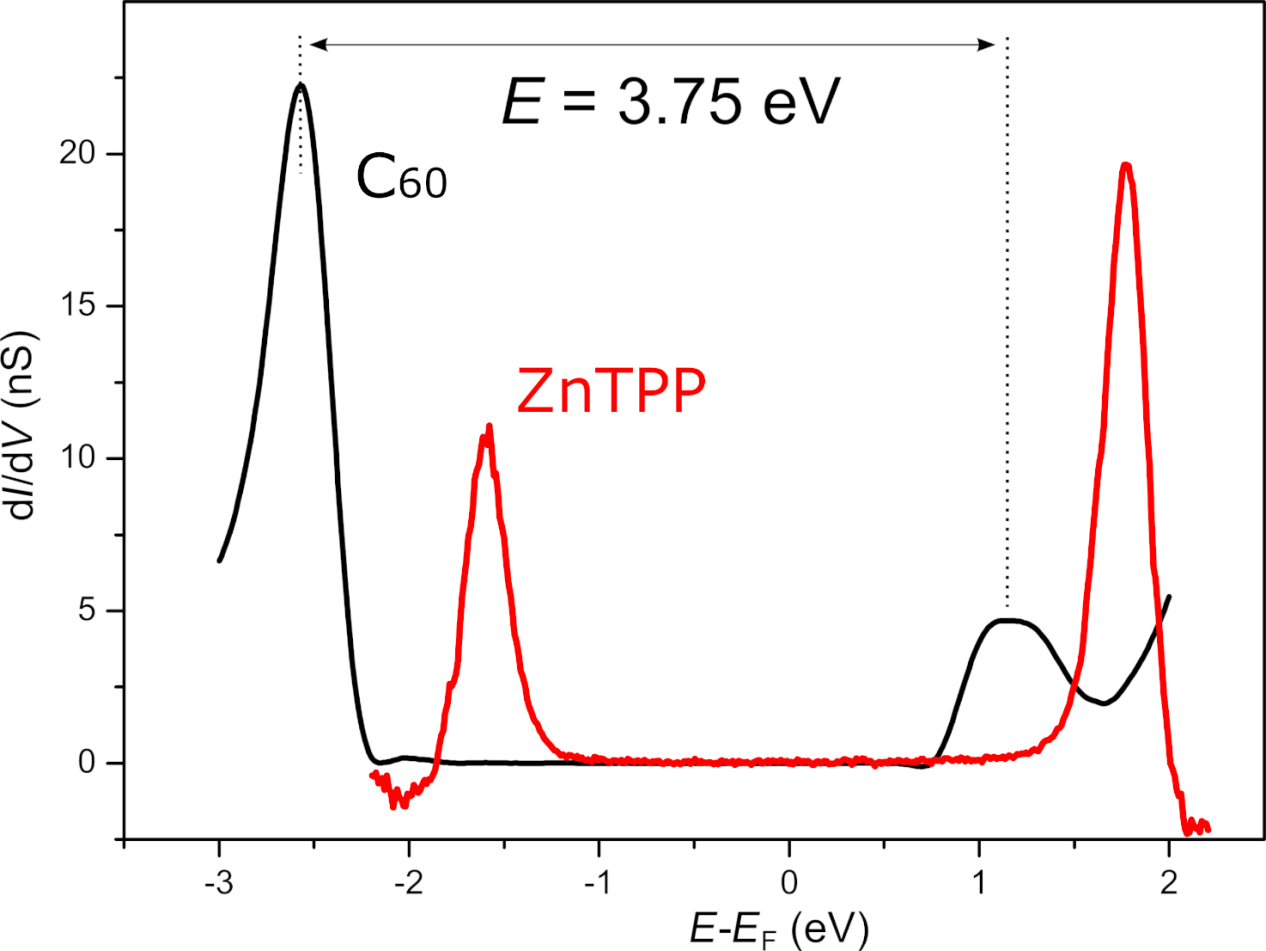
Scanning tunneling spectrum acquired at constant tip–surface separation (open feedback loop) on the C_60_/ZnTPP/Fe(001)–*p*(1 × 1)O system (black) and on the ZnTPP/Fe(001)–*p*(1 × 1)O surface (red). The black curves have been obtained by averaging 30 single spectra taken on equivalent C_60_ domains. The set point before the acquisition of the spectra was set to *V* = 1.5 V and *I* = 1 nA.

In order to determine the HOMO–LUMO gap of the C_60_ film, STS measurements have been acquired for both negative and positive bias to investigate the filled and empty electronic states, respectively. Figure 4 shows STS spectra acquired on the ZnTPP/Fe(001)–*p*(1 × 1)O surface (red) and on the C_60_/ZnTPP/Fe(001)–*p*(1 × 1)O system (black). The STS measurements acquired on ZnTPP/Fe(001)–*p*(1 × 1)O are in excellent agreement with those published in [43]. The STS curve referring to C_60_/ZnTPP/Fe(001)–*p*(1 × 1)O has been obtained by averaging several spectra acquired on equivalent C_60_ domains. We acquired also spectra in different locations of single C_60_ molecules, but not significant differences with a well-defined trend were observed. In the negative energy range (filled electronic states) a strong resonance centered at about −2.60 eV is present, which we attribute to HOMO states, in excellent agreement with UPS measurements (−2.56 eV). In the positive energy range (empty states) of the STS spectrum the LUMO peak is visible at 1.15 eV, resulting in an electronic gap equal to 3.75 eV.

**Figure 5:**
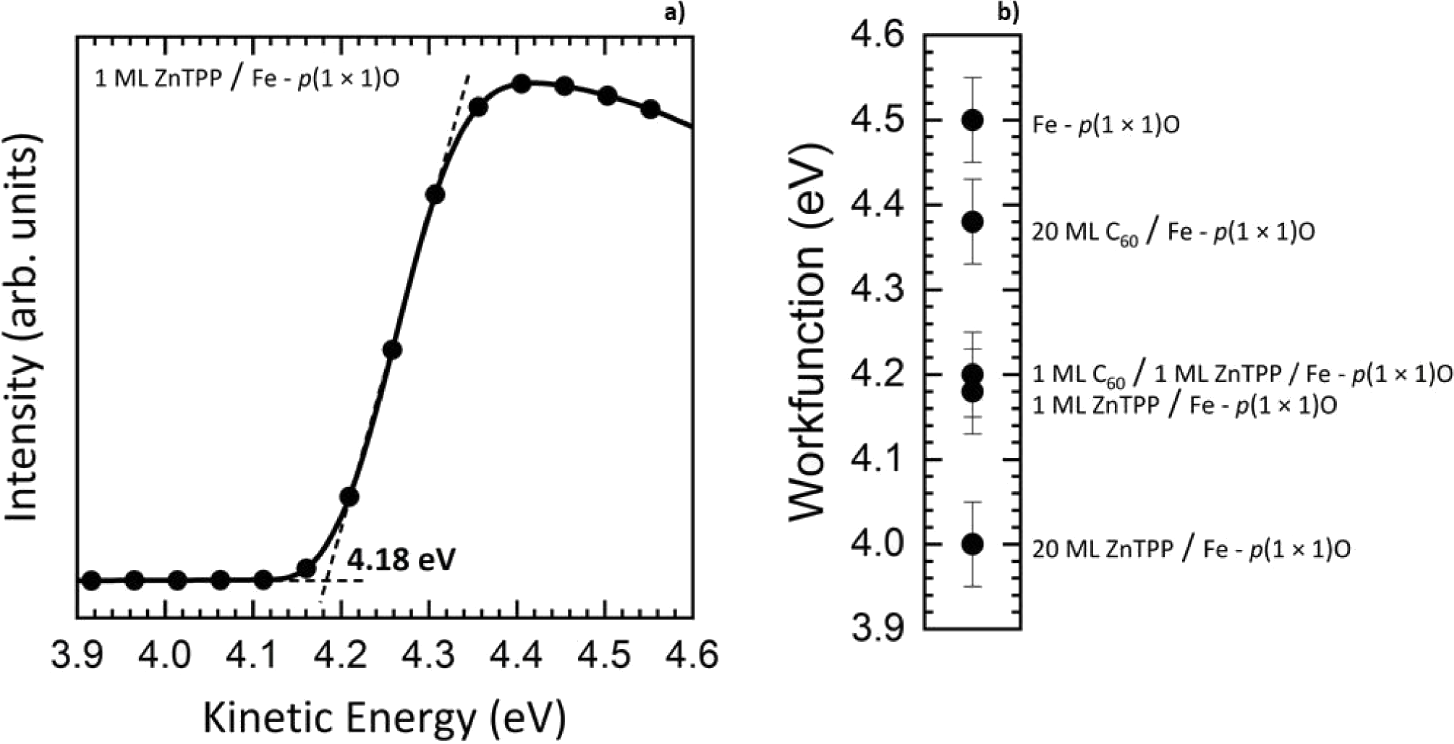
(a) Work function acquired on the system 1 ML ZnTPP/Fe(001)–*p*(1 × 1)O. The two dashed lines indicate how the onset of the curve is determined. The 10 eV offset due to the bias applied to the system has been subtracted. (b) Summary of the values of the work functions acquired on the system Fe(001)–*p*(1 × 1)O at different molecular coverages.

Finally, work function measurements have been performed to evaluate the charge transfer between the different layers constituting the heterostructure. Generally, electron transfer from the substrate (overlayer) to the overlayer (substrate) induces an increase (decrease) of the work function with respect to the bare surface. For the work function measurements, the sample has been biased with a voltage of 10 V to detect the onset of the secondary electrons. The onset position is determined as the intersection of the zero-current line and the tangent to the rising edge of the data. Figure 5a displays a typical UPS spectrum in an energy range straddling the high-binding-energy cutoff of the secondary electrons, which we exploit for the evaluation of the work function for 1 ML ZnTPP/Fe(001)–*p*(1 × 1)O. The 10 eV offset due to the bias applied to the system has already been accounted for. In Figure 5b, the evolution of the work function for the different samples is presented. Starting from the bare substrate, the work function is reduced by about 0.3 eV after the deposition of 1 ML of ZnTPP, in agreement with previous measurements [42]. Such a decrease has been ascribed to charge transfer from ZnTPP to the Fe(001)–*p*(1 × 1)O substrate. When 1 ML of C_60_ is added, the variation of the work function is within the experimental error, indicating a negligible charge transfer on the surface region upon C_60_ adsorption.

The electronic properties of C_60_ adsorbed on ZnTPP deserve a deeper discussion. We recall that the difference between the energies of LUMO and HOMO orbitals of C_60_ at equilibrium is about γ = 1.6 eV, as determined experimentally [57] and theoretically [58]. However, the difference between the electron affinity and the ionization potential measured on isolated C_60_ (in the gas phase) is about *E*_s_ = 4.95 eV [57], considerably higher than γ. This discrepancy is given by the fact that the ionization potential (electron affinity) is not simply the difference between the vacuum level and the HOMO (LUMO) energies of C_60_ at equilibrium, because an extra energy is required to remove (add) an electron from (to) the neutral molecule. Therefore, the gap measured with electron-based spectroscopic experiments is *E* = γ + *U*, where *U* is the on-site Coulomb energy [57]. The *U* term accounts for the fact that, when occupied states are probed, an electron is removed from the molecule, therefore the measured spectrum is not representative of the neutral but of the positively charged molecule. Similarly, when unoccupied states are probed, an electron is injected in the molecule and the system is negatively charged. For isolated C_60_ molecules, the charging energy is *U*_s_ = *E*_s_ − γ *=* 3.35 eV. In [57], Esper et al. measured γ by performing PES on C_60_ films highly doped with K. In this case, the LUMO orbitals were completely filled, therefore the charging energy was the same when HOMO and LUMO states were probed and the difference between the LUMO and HOMO energies was independent from *U*.

Generally, when C_60_ is adsorbed on a substrate, the *U* term is drastically reduced by the electrostatic screening provided by the metallic or molecular support. In the former case, when an electron is added or removed from C_60_, the charged molecule is screened by an opposite image charge underneath the metal surface, while, in the latter case, the screening is provided by electric dipoles induced on the organic substrate. In order to evaluate the coupling between C_60_ and the substrate, it is useful to quantify the reduction of the electronic gap *E* (or equivalently of the *U* term) with respect to that of the isolated molecule. In the case of the (111) surface of face-centered cubic bulk C_60_, the measured electronic gap is *E*_b_ = 3.50 eV [23]. Therefore, the charging energy is *U*_b_ = 1.90 eV. Defining Δ*U* as the variation of the Coulomb energy with respect to isolated C_60_, in the case of bulk C_60_, it is found Δ*U* = *U*_b_ − *U*_s_ = −1.45 eV. Such a decrease of *U* can be ascribed to the polarization of the nine molecules surrounding each C_60_ located at the surface, six belonging to the topmost layer and three to the second layer. By considering an equal contribution for each molecule, every C_60_ provides a screening of about Δ*U* = −0.16 eV.

Starting from this observation, it is possible to evaluate the screening provided by the Fe(001)–*p*(1 × 1)O and ZnTPP/Fe(001)–*p*(1 × 1)O substrates on the C_60_ film (see Table 1). To this aim, we can assume that Δ*U* is the sum of two contributions, the first one due to the screening provided by six surrounding C_60_ molecules (Δ*U*_surf_) and the second one provided by the substrate (Δ*U*_sub_). As for Δ*U*_surf_, we consider for each sample the same value as found in bulk C_60_(111), because C_60_ forms a hexagonal lattice also on top of the other substrates. In the case of C_60_/Fe(001)–*p*(1 × 1)O, it is found Δ*U*_sub_ = −0.59 eV. Therefore, the oxygen-passivated Fe(001) surface provides a higher screening with respect to a fullerene substrate. In contrast, for the C_60_/ZnTPP/Fe(001)–*p*(1 × 1)O sample, it is found Δ*U*_sub_ = −0.24 eV, indicating a very low screening induced by the porphyrin buffer layer, even with respect to that provided by a substrate of bulk C_60_.

**Table 1:** Electronic coupling of C_60_ with the Fe(001)–*p*(1 × 1)O and ZnTPP/Fe(001)–*p*(1 × 1)O substrates. *E* is the energy gap measured by electron-based spectroscopic techniques. *U* = *E* − γ is the on-site Coulomb energy, where γ = 1.6 eV is the HOMO–LUMO energy difference at equilibrium. Δ*U*_surf_ and Δ*U*_sub_ are variations of *U* with respect to the value of isolated C_60_ due to the topmost layer and the substrate, respectively.

System	*E* (eV)	*U* (eV)	Δ*U*_surf_ (eV)	Δ*U*_sub_ (eV)

isolated C_60_ [42]	4.95	3.35	0	0
C_60_ bulk [22]	3.50	1.90	−0.96	−0.49
C_60_/Fe(001)–*p*(1 × 1)O [22]	3.40	1.80	−0.96	−0.59
C_60_/ZnTPP/Fe(001)–*p*(1 × 1)O	3.75	2.15	−0.96	−0.24

## Conclusion

In conclusion, the electronic and morphological properties of a single layer of C_60_ deposited on a ZnTPP/Fe(001)–*p*(1 × 1)O substrate have been investigated. The ZnTPP buffer layer promotes the surface diffusion of C_60_ and the growth of a crystalline film at room temperature. The large HOMO–LUMO gap and the negligible charge transfer at the interface indicate that C_60_ is electronically decoupled from the substrate. The C_60_/ZnTPP/Fe(001)–*p*(1 × 1)O multilayer represents a paradigmatic system in which the electronic properties of a single layer of fullerene in close proximity to a metallic substrate are preserved.
